# Nickel–Cobalt Layered Double Hydroxide Nanosheet-Decorated 3D Interconnected Porous Ni/SiC Skeleton for Supercapacitor

**DOI:** 10.3390/molecules29235664

**Published:** 2024-11-29

**Authors:** Han-Wei Chang, Chia-Hsiang Lee, Shih-Hao Yang, Kuo-Chuang Chiu, Tzu-Yu Liu, Yu-Chen Tsai

**Affiliations:** 1 Department of Chemical Engineering, National United University, Miaoli 360302, Taiwan; m1214014@o365.nuu.edu.tw; 2 Pesticide Analysis Center, National United University, Miaoli 360302, Taiwan; 3 Department of Chemical Engineering, National Chung Hsing University, Taichung 40227, Taiwan; g112065059@mail.nchu.edu.tw; 4 Material and Chemical Research Laboratories, Industrial Technology Research Institute, Hsinchu 310401, Taiwan; ckc@itri.org.tw (K.-C.C.); jill.t.y.liu@itri.org.tw (T.-Y.L.)

**Keywords:** 3D Ni/SiC skeleton, nickel–cobalt layered double hydroxide, supercapacitor, pseudocapacitance, EDLC

## Abstract

In this study, a three-dimensional (3D) interconnected porous Ni/SiC skeleton (3D Ni/SiC) was synthesized by binder-free hydrogen bubble template-assisted electrodeposition in an electrolyte containing Ni^2+^ ions and SiC nanopowders. This 3D Ni/SiC skeleton served as a substrate for directly synthesizing nickel–cobalt layered double hydroxide (LDH) nanosheets via electrodeposition, allowing the formation of a nickel–cobalt LDH nanosheet-decorated 3D Ni/SiC skeleton (NiCo@3D Ni/SiC). The multiscale hierarchical structure of NiCo@3D Ni/SiC was attributed to the synergistic interaction between the pseudocapacitor (3D Ni skeleton and Ni–Co LDH) and electrochemical double-layer capacitor (SiC nanopowders). It provided a large specific surface area to expose numerous active Ni and Co sites for Faradaic redox reactions, resulting in an enhanced pseudocapacitance. The as-fabricated NiCo@3D Ni/SiC structure demonstrated excellent rate capability with a high areal capacitance of 1565 mF cm^−2^ at a current density of 1 mA cm^−2^. Additionally, symmetrical supercapacitor devices based on this structure successfully powered commercial light-emitting diodes, indicating the potential of as-fabricated NiCo@3D Ni/SiC in practical energy storage applications.

## 1. Introduction

Electrochemical energy storage devices like fuel cells, batteries, and supercapacitors are widely used in mobile electronics and systems (e.g., phones, laptops, electric vehicles, and electric grid integration), addressing critical energy issues such as resource depletion, climate change, and environmental harm [1,2,3]. Among energy storage devices, supercapacitors are notable for their high specific capacitance, excellent rate capability, and cycling stability, which make them ideal for next-generation energy storage. They are classified into electrochemical double-layer capacitors (EDLCs) and pseudocapacitors based on the charge storage mechanism. EDLCs store charges via a non-Faradaic electrostatic process at electrode–electrolyte interfaces. Semiconductor and cermet materials (such as silicon, titanium nitride, titanium dioxide, and silicon carbide) are ideal for EDLC electrodes [3,4,5,6,7]. Studies suggest rational design strategies for EDLC-type hierarchical architectures using semiconductor and cermet materials. Integrating these materials with conductive elements (metal or carbon-based) creates an interconnected conductive skeleton, presenting new opportunities for energy storage [8,9,10,11,12]. This design greatly enhances the specific surface area and electrical conductivity of electrode materials, improving supercapacitor capacitive performance.

SiC-based materials with a tunable bandgap possess optical, thermal, mechanical, and chemical properties, making them ideal for supercapacitor electrodes. Recent studies have demonstrated that 3D interconnected skeletons, created by combining SiC with conductive materials, offer a conductive network with high potential for supercapacitor applications [13,14,15,16]. These conductive networks support the direct growth or deposition of active materials by controlling nucleation and growth processes, acting as conductive bridges to shorten ion/electron transport distances, accelerate ion diffusion, and enhance electrode kinetics, thus significantly improving supercapacitor performance.

Pseudocapacitors store charges via Faradaic reactions on or near redox species’ surfaces. Transition metal compounds, known for their excellent electrical conductivities, diverse crystalline structures, and valence states, are ideal as electrode materials in pseudocapacitors. Compounds based on Ni, Co, Cu, and Fe boast high theoretical specific capacities due to the presence of numerous electrochemical redox-active sites, a highly crystalline structure, and valence state transitions, which facilitate high-pseudocapacitive-energy storage [17,18,19]. For the enhancement of energy storage in supercapacitors, transition metal compounds have been directly grown on hierarchical architectures with conformal contacts to obtain multiscale hierarchical architectures for use as binder-free supercapacitors. Furthermore, owing to the 3D interconnected conductive network, there is a high degree of interfacial contact between the electroactive materials and high specific surface area, and it leads to enhanced mass/charge transfer at the electrode–electrolyte interface. So far, Ni-based 3D conductive architectures have attracted great attention as promising electrode materials in supercapacitors, since they have high theoretical specific capacitance, excellent electrical conductivity, great thermal/chemical stability, and a low cost [20]. Different methods have been reported to prepare a Ni-based 3D conductive architecture and the subsequent direct growth of various active materials on it or for binder-free electrode preparation. Currently, the methods for preparing a Ni-based 3D conductive architecture include hydrothermal methods [21], chemical bath deposition [22], microwave-assisted deposition [23], laser-assisted deposition [24], and electrochemical deposition [25]. In our previous study [26], a Ni-based 3D conductive architecture was synthesized using electrochemical deposition; such a 3D conductive substrate could offer more available contact areas and anchoring active sites for the nucleation and conformal growth of active materials to construct advanced 3D interconnected architectures. The exceptional electronic conductivity and hierarchical interconnected 3D conductive architecture would result in significant enhancement of electrochemical kinetics.

A hybrid supercapacitor assembled with both EDLC- and pseudo-type electrode materials offers one of the most effective ways to improve the electrochemical capacitive performance of a supercapacitor. The synergistic effects boost the capacitive performance of the supercapacitor remarkably [27,28,29]. EDLC supercapacitors based on semiconductor and cermet materials possess high electron mobility, good physicochemical stability, and excellent charge–discharge cycling stability. When they transform from bulk to nanoscale, it is expected that these nanoscale structures could augment the surface area to afford more EDLCs, resulting in the ideal capacitive behavior. Pseudocapacitors based on transition metal compounds enhance the utilization of redox-active sites participating in the redox reaction, providing additional pseudocapacitance. A previous study reported that the combination of semiconductor and cermet materials as EDLC-type electrode materials and transition metal compounds as pseudo-type electrode materials was developed to achieve high supercapacitor performance [27,30,31].

In this study, nickel–cobalt layered double hydroxide (LDH) nanosheets were uniformly grown on a 3D interconnected porous Ni/SiC skeleton (consisting of numerous interconnected skeletons), which served as a support for transition metal compounds and a conductive bridge for a multiscale hierarchical structure. This interconnected architecture promoted efficient electronic conduction and ionic diffusion, improving supercapacitor performance.

## 2. Results and Discussion

**Figure 1:**
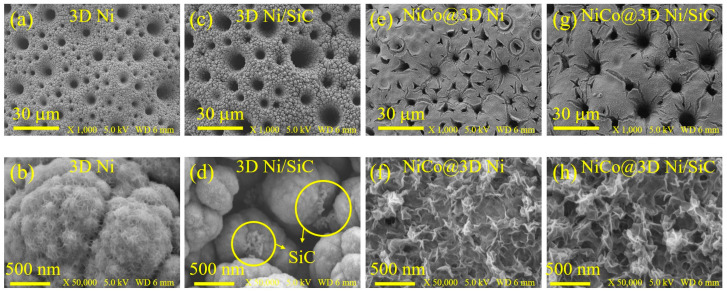
Low- (**a**,**c**,**e**,**g**) and high-magnification (**b**,**d**,**f**,**h**) FESEM images of (**a**,**b**) 3D Ni, (**c**,**d**) 3D Ni/SiC, (**e**,**f**) NiCo@3D Ni, and (**g**,**h**) NiCo@3D Ni/SiC.

**Figure 2:**
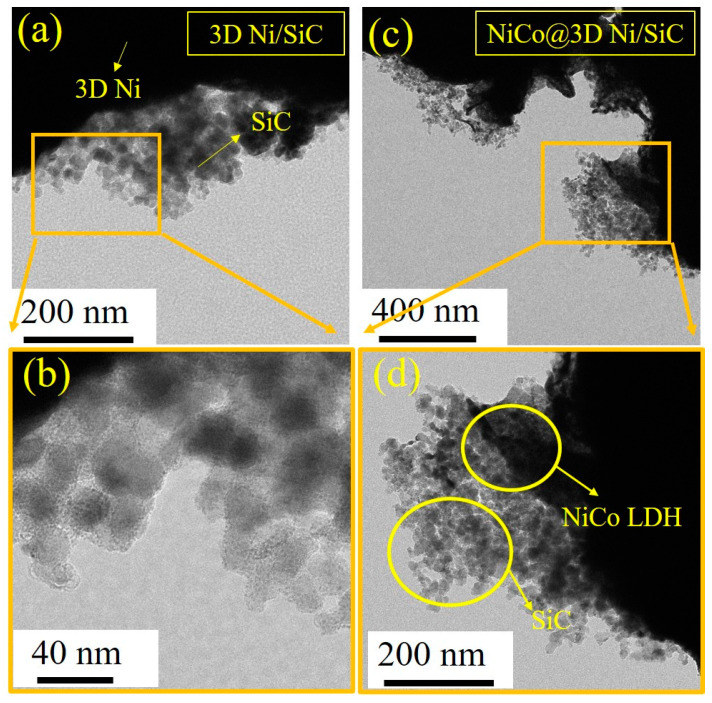
TEM images of (**a**,**b**) 3D Ni/SiC and (**c**,**d**) NiCo@3D Ni/SiC.

Surface morphologies of the 3D Ni, 3D Ni/SiC, NiCo@3D Ni, and NiCo@3D Ni/SiC samples were examined using field-emission scanning electron microscopy (FESEM) and transmission electron microscopy (TEM). Figure 1a,b present low- and high-magnification FESEM images of 3D Ni, revealing its porous and interconnected network structure with pore diameters ranging from sub-micrometers to several micrometers. These images confirm the successful synthesis of interconnected porous 3D Ni using hydrogen bubble templates. For the electrodeposition of 3D Ni/SiC, SiC nanopowder (30 nm) was incorporated into the electrolyte, with the resulting mixture serving as the supporting electrolyte. The FESEM images in Figure 1c,d reveal dense, well-distributed SiC nanoclusters (ranging from tens to hundreds of nanometres) (marked with yellow circles) within a 3D interconnected porous Ni, demonstrating the successful incorporation of SiC nanopowder. The 3D Ni/SiC exhibited an EDLC-type hierarchical structure, highlighting the synergy between the porous Ni skeleton and SiC nanoclusters. This structure can act as a substrate with a large specific surface area for the growth of other active materials. The 3D Ni/SiC provided a sufficiently large electrode–electrolyte interface area to the reduce ion/electron transport distance, enhancing supercapacitor performance. Three-dimensional Ni and 3D Ni/SiC were used as substrates and current collectors for the growth of nickel–cobalt LDH nanosheets via electrodeposition. After electrodeposition, in situ uniform growth of dense and interconnected nickel–cobalt LDH occurred on the skeleton surfaces of 3D Ni and 3D Ni/SiC, leading to the formation of NiCo@3D Ni (Figure 1e,f) and NiCo@3D Ni/SiC (Figure 1g,h). The synthesized NiCo@3D Ni and NiCo@3D Ni/SiC consisted of ultrathin nanosheets, and partial aggregation led to a uniform dispersion of 2D Ni–Co nanosheet clusters. Figure 2 shows TEM images of 3D Ni/SiC and NiCo@3D Ni/SiC. Figure 2a,b show well-distributed SiC nanoclusters (approximately 30 nm) within the bulk 3D Ni skeleton, confirming the successful incorporation of SiC in the porous 3D Ni. TEM images of NiCo@3D Ni/SiC are shown in Figure 2c,d, and they strongly suggest that the 3D Ni/SiC allowed uniform incorporation of 2D Ni-Co nanosheets to synthesize NiCo@3D Ni/SiC (marked with yellow circles). The FESEM and TEM images showed that the successful incorporation of 2D Ni-Co nanosheets over the 3D Ni/SiC skeleton has its own unique structural characteristics, which could afford the NiCo@3D Ni/SiC maximal exposed active sites and further provide extra pseudocapacitance.

**Figure 3:**
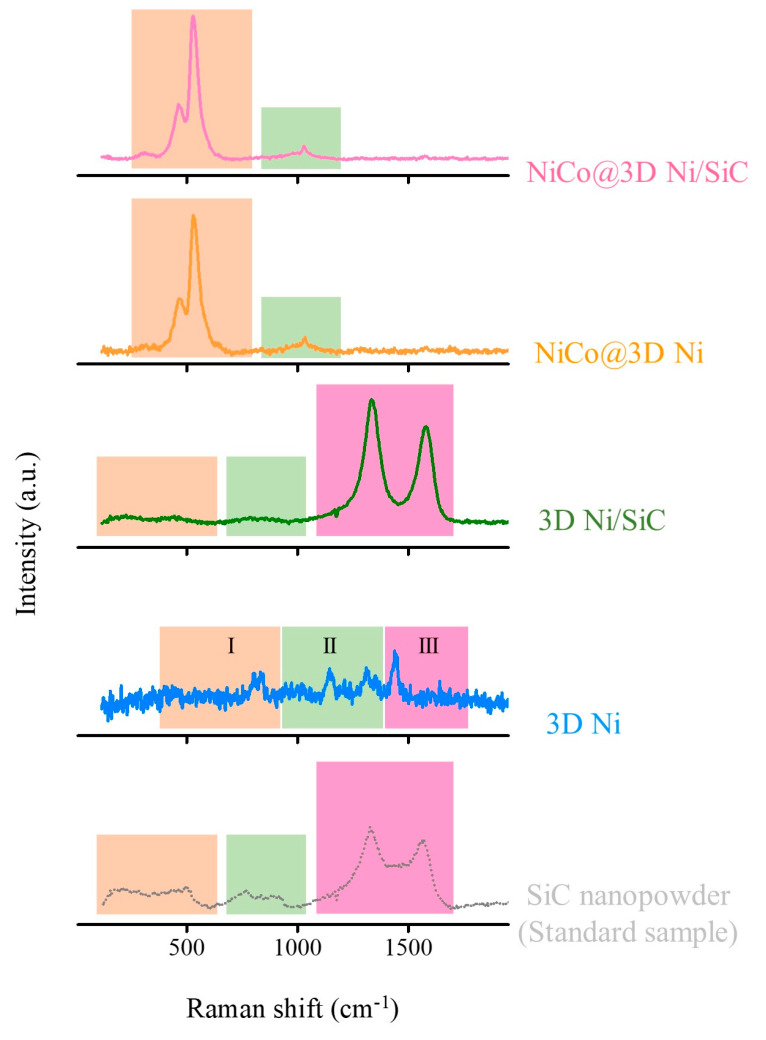
Raman spectra of SiC nanopowder, 3D Ni, 3D Ni/SiC, NiCo@3D Ni, and NiCo@3D Ni/SiC.

Raman spectroscopy was used to verify the presence of SiC and nickel–cobalt LDH in the 3D Ni, 3D Ni/SiC, NiCo@3D Ni, and NiCo@3D Ni/SiC samples. The structure and phase composition of these samples and commercial β-phase SiC nanopowder were assessed using Raman spectroscopy (Figure 3). The Raman spectrum of 3D Ni exhibited four characteristic Raman peaks originated from NiO within the range of 700 to 1500 cm^−1^, which can be generally separated into three different regions marked with different colors, namely, region I (Raman shift between 300 and 700 cm^−1^), region II (Raman shift between 700 and 1200 cm^−1^), and region III (Raman shift between 1200 and 1500 cm^−1^) were assigned to first-order phonon modes, second-order phonon modes, and magnetic ordering, respectively [32]. In order to further prove the existence of SiC in the 3D Ni/SiC sample, commercial β-phase SiC nanopowder was used as the standard sample to verify the successful synthesis of the 3D Ni/SiC sample. In commercial β-phase SiC nanopowder, the broad Raman spectrum confirmed the presence of SiC with relatively low crystallinity and the spectrum could be divided into three different regions (Raman shift between 200 and 500 cm^−1^, 700 and 950 cm^−1^, and 1400 and 1600 cm^−1^) marked with different colors. The first broad band originates from the mixed characteristic Raman peaks of transverse (at around 210 cm^−1^) and longitudinal (at around 524 cm^−1^) acoustic phonon modes of SiC or amorphous Si. The second broad band from 750 to 950 cm^−1^ could be attributed to the mixed characteristic Raman peaks of transverse (at 776 cm^−1^) and longitudinal (at around 934 cm^−1^) optical phonon modes of β-phase SiC, and the third broad band corresponds to the disorder (D band) and perfect (G band) modes of the sp^2^/sp^3^ coordination of carbon [33,34]. Excess carbon was detected in the commercial β-phase SiC nanopowder, likely due to the stoichiometry variations in the SiC synthesis process [35]. The Raman spectrum of 3D Ni/SiC showed characteristic peaks identical to β-phase SiC, confirming the presence of β-phase SiC and its incorporation into the 3D Ni structure with a hierarchical porous skeleton. After Ni–Co LDH growth, both the NiCo@3D Ni and NiCo@3D Ni/SiC samples exhibited intense Raman peaks at approximately 464 and 533 cm^−1^ (marked with light orange color), corresponding to Ni–O and Co–O vibrational modes. Additionally, a relatively low-intensity broad Raman peak appeared at 1040 cm^−1^, which was attributed to the symmetrical stretching vibration of nitrate anions [36]. It is also noticed that no NiO and SiC characteristic Raman peaks appear in the NiCo@3D Ni and NiCo@3D Ni/SiC samples due to complete nickel–cobalt LDH film covering of the 3D Ni and 3D Ni/SiC substrate. The formation of nuclei at the metal surface covers part of the active sites. The Raman spectrum provided strong evidence of the 2D Ni-Co LDH structure containing intercalated nitrate anions. The intercalation of nitrate anions as interlayer spacers can increase the basal spacing of 2D Ni-Co LDH nanosheets, resulting in the formation of a convenient charge transfer diffusion path that would boost the electrochemical performance.

**Figure 4:**
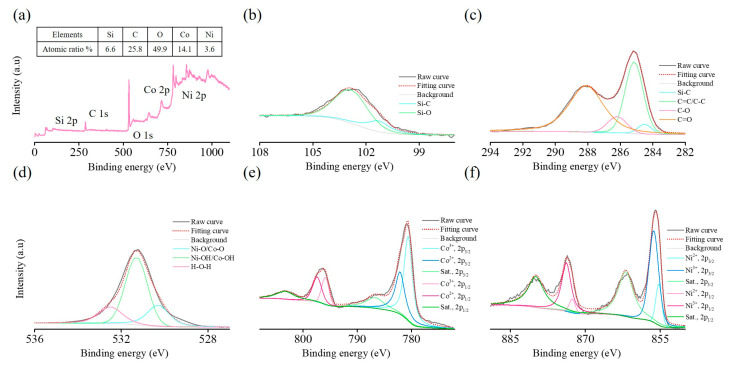
XPS spectra of NiCo@3D Ni/SiC: (**a**) XPS spectrum of survey scan and high-resolution XPS spectrum of (**b**) Si 2p, (**c**) C 1s, (**d**) O 1s, (**e**) Co 2p, and (**f**) Ni 2p. The insert table in (**a**) is the atomic percentage (%) of the elements.

To obtain detailed surface structure and compositional information for evaluating capacitive performance after incorporating nickel–cobalt LDH into the 3D porous Ni/SiC skeleton, we analyzed the surface elemental composition of NiCo@3D Ni/SiC using X-ray photoelectron spectroscopy (XPS), as shown in Figure 4. The full XPS survey spectrum in Figure 4a indicates that Si, C, O, Co, and Ni are the primary components present in the as-prepared NiCo@3D Ni/SiC. And, the atomic percentage (%) of the elements for NiCo@3D Ni/SiC are given in the insert table in Figure 4a, which displays 6.6, 25.8, 49.9, 14.1, and 3.6 atomic percentages of Si, C, O, Co, and Ni elements. High-resolution XPS spectra were employed to characterize the elemental composition and valence state of NiCo@3D Ni/SiC. Figure 4b presents a high-resolution Si 2p XPS spectrum, split into two main peaks at 100.3 and 103.2 eV, indicating Si–C and Si–O bonds. The Si–O bond likely formed upon air exposure to or oxidation by SiOx during electrodeposition [37,38]. Figure 4c shows the C 1s XPS spectrum, decomposed into four components: Si–C (284.2 eV), C=C/C–C (285.2 eV), C–O (286.0 eV), and C=O (289.1 eV). XPS analysis of the Si 2p and C 1s spectra confirmed that hydrogen bubble template-assisted electrodeposition produced a composite of SiC species within the 3D interconnected porous Ni/SiC skeleton.

Figure 4d presents the O 1s XPS spectrum, which can be deconvoluted into three peaks at 530.2, 531.1, and 532.8 eV, corresponding to lattice oxygen (Ni–O/Co–O), hydroxide ions (Ni–OH/Co–OH), and adsorbed water (H–O–H), respectively [39,40]. Figure 4e displays the high-resolution Co 2p XPS spectrum, featuring two Co 2p_3/2_–Co 2p_1/2_ spin–orbit doublets and their shake-up satellite peaks at approximately 786.7 and 803.3 eV. The 2p_3/2_ and 2p_1/2_ spin–orbit coupling patterns are fitted with peaks at 780.5 eV (or 782.2 eV) for Co 2p_3/2_ and 795.7 eV (797.4 eV) for Co 2p_1/2_, indicating the presence of Co^3+^/Co^2+^ [41]. The Ni 2p XPS spectrum in Figure 4f displays two spin–orbit doublets, Ni 2p_1/2_ and Ni 2p_3/2_, with shake-up satellite peaks at approximately 861.8 and 879.9 eV. To identify Ni species, the Ni 2p_3/2_ and Ni 2p_1/2_ doublets were divided into two fitting peaks representing mixed valence states Ni^2+^/Ni^3+^. Peaks at 855.2 eV (856.5 eV) and 872.5 eV (873.9 eV) in the Ni 2p_3/2_ and Ni 2p_1/2_ doublets correspond to Ni^2+^ (Ni^3+^) [42]. The results confirmed that the presence of Co^2+^/Co^3+^ and Ni^2+^/Ni^3+^ in NiCo@3D Ni/SiC likely enhanced its pseudocapacitive performance.

**Figure 5:**
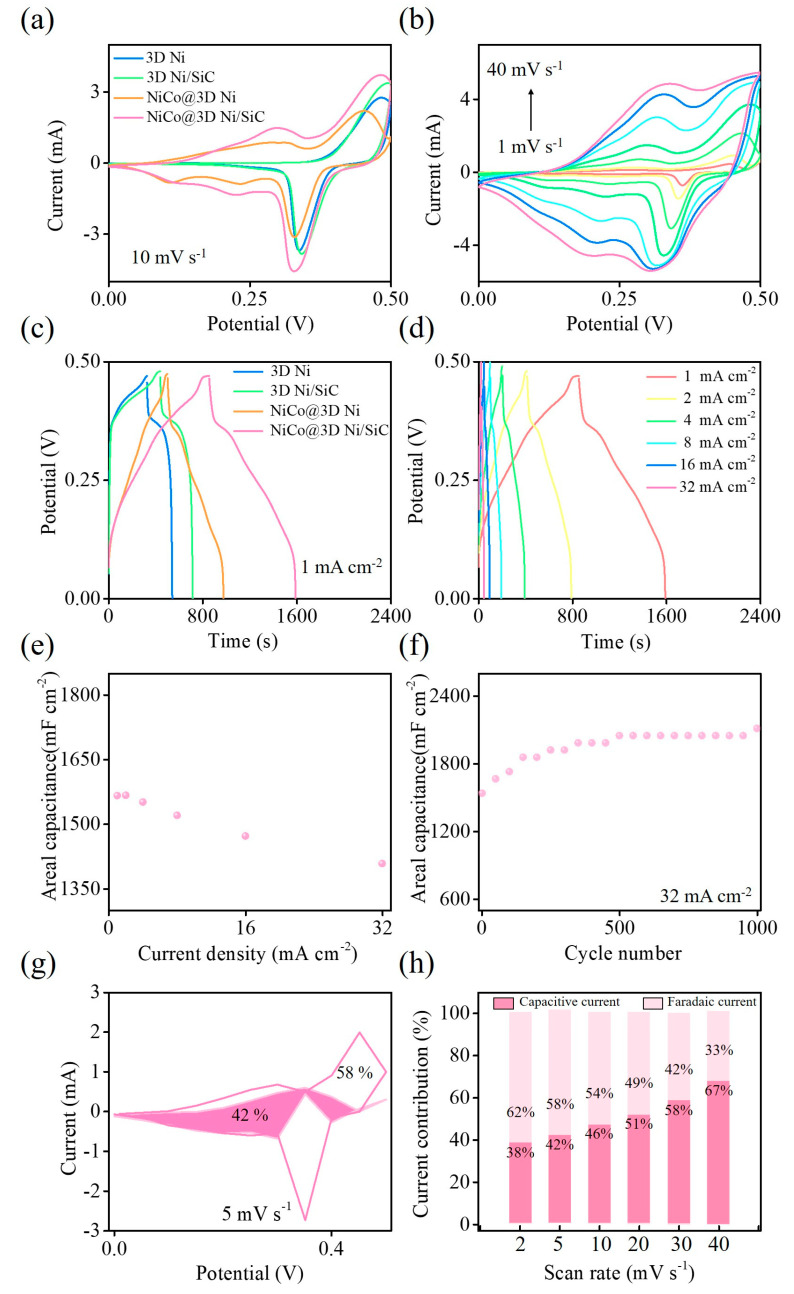
(**a**) CV (scan rate of 10 mV s^−1^) and (**c**) GCD (current density of 1 mA cm^−2^) of 3D Ni, 3D Ni/SiC, NiCo@3D Ni, and NiCo@3D Ni/SiC. (**b**) CV of NiCo@3D Ni/SiC at different scan rates from 1 to 40 mV s^−1^. (**d**) GCD of NiCo@3D Ni/SiC at different current densities from 1 to 32 mA cm^−2^ in a three-electrode system with a potential window between 0 and 0.5 V versus Ag/AgCl in 1 M KOH solution. (**e**) Current densities vs. specific areal capacitance from (**d**) results. (**f**) Cycling test of NiCo@3D Ni/SiC at a current density of 32 mA cm^−2^. (**g**) The contribution percentages of diffusion-controlled Faradaic and surface-controlled capacitive behaviors of NiCo@3D Ni/SiC at a scan rate of 5 mV s^−1^. (**h**) Contribution percentages at various scan rates.

Cyclic voltammetry (CV) and galvanostatic charge–discharge (GCD) tests were conducted using a three-electrode system to assess the capacitive behavior of the 3D Ni, 3D Ni/SiC, NiCo@3D Ni, and NiCo@3D Ni/SiC samples. Both tests were performed in the potential window of 0.0 to 0.5 V with 1 M KOH aqueous solution as the electrolyte. Figure 5a compares the CV curves of the 3D Ni, 3D Ni/SiC, NiCo@3D Ni, and NiCo@3D Ni/SiC samples at a scan rate of 10 mV s^−1^. The redox peaks at 0.4–0.5 V, shown by the 3D Ni and 3D Ni/SiC samples, indicate that the Faradiac redox reaction can be attributed to a reversible reaction involving Ni^2+^/Ni^3+^ transitions. The area under the CV curve of the 3D Ni/SiC sample is larger than that of the 3D Ni sample, confirming the synergistic effect of pseudocapacitance from the 3D Ni skeleton and electrochemical double-layer capacitance from the SiC nanopowders. The heterostructure of the 3D interconnected porous Ni/SiC skeleton offered more active sites for rapid Faradaic redox reactions and electrostatic charge storage in the EDLC, resulting in significantly higher capacitance than the 3D Ni sample. The as-synthesized 3D Ni and 3D Ni/SiC samples acted as interconnected nanoscale frameworks for the growth of nickel–cobalt LDH nanosheets, revealing redox couples in the CV curves. The CV curves of NiCo@3D Ni and NiCo@3D Ni/SiC showed a larger integration area and well-defined higher reversible redox peaks, indicating the redox pseudocapacitance contribution of the electrodes. The reversible redox reaction originated due to the transition of Ni^2+^/Ni^3+^, Co^2+^/Co^3+^, and Co^3+^/Co^4+^ on the electrode surface, evidencing the coexistence of Ni and Co elements [43,44]. The well-defined redox couples suggested rapid kinetics of Faradaic redox reactions and ion diffusion through the electrodes, indicating the highly pseudocapacitive feature of the electrodes. The integration area associated with the CV curve of the NiCo@3D Ni/SiC sample was larger than that of the NiCo@3D Ni sample, indicating a significantly higher electrochemical capacitance. This higher capacitance facilitated the formation of multiple redox-active sites and was due to the interconnected hierarchical nickel–cobalt LDH nanosheets and conductive 3D Ni/SiC skeleton. CV curves of the NiCo@3D Ni/SiC sample, obtained at various scan rates (Figure 5b), showed a noticeable increase in peak current as the scan rate increased from 1 to 40 mV s^−1^, while the curve shapes remained almost unchanged. This confirmed the sample’s rate capability and superior pseudocapacitive behavior.

GCD tests were conducted to determine the specific areal capacitance of the 3D Ni, 3D Ni/SiC, NiCo@3D Ni, and NiCo@3D Ni/SiC samples. The GCD curves at a current density of 1.0 mA cm^−2^ are shown in Figure 5c. All the samples exhibited quasi-triangular symmetrical GCD curves with plateaus, indicating redox couples consistent with CV results. This distribution is due to the synergistic effect of pseudocapacitance (from Ni and Co) and electrochemical double-layer capacitance (from SiC nanopowders). Areal capacitance was calculated from the GCD curve using *C* = (*I*Δ*t*)/Δ*V* (where *C* is the areal capacitance (in millifarads per square centimeter), *I* is the current density (in milliamperes per square centimeter), Δ*t* is the discharge time (in seconds), and Δ*V* is the voltage change during discharge (in volts)). The 3D Ni/SiC (577 mF cm^−2^)/NiCo@3D Ni/SiC (1565 mF cm^−2^) samples showed higher specific areal capacitance than the 3D Ni (457 mF cm^−2^)/NiCo@3D Ni (1002 mF cm^−2^) samples. The incorporation of SiC reduced contact resistance and increased the space between the 3D interconnected Ni skeleton and hierarchical nickel–cobalt LDH nanosheets. These features allowed the formation of a large contact interface and more active sites between the electrolyte and electrode, resulting in a higher capacitance. The GCD curves of NiCo@3D Ni/SiC at various current densities from 1.0 to 32 mA cm^−2^ are presented in Figure 5d. As the current density increased, the shapes of the GCD curves were well reproducible, and the charge–discharge time decreased. The values of the areal capacitance at current densities of 1.0–32 mA cm^−2^ were 1565, 1567, 1551, 1520, 1472, and 1408 mF cm^−2^. Even at a high current density of 32 mA cm^−2^, 90% of the initial specific areal capacitance was retained, which demonstrated the outstanding rate capability of the sample (Figure 5e). To further evaluate the capacitance stability of NiCo@3D Ni/SiC, charge–discharge cycling tests were performed on the NiCo@3D Ni/SiC sample for 1000 cycles using a three-electrode system with a KOH electrolyte at a high current density of 32 mA cm^−2^ (Figure 5f). The areal capacitance of the NiCo@3D Ni/SiC sample gradually increased with the number of cycles, and a capacitance of approximately 2072 mF cm^−2^ was retained, indicating exceptional long-term capacitance stability.

The detailed contributions of charge storage mechanisms in the NiCo@3D Ni/SiC sample were conducted from the CV curves obtained from different scan rates ranging from 2 to 40 mV s^−1^. CV results by calculating from different scan rates (Figure 5a) may allow us to evaluate the relative contributions of the diffusion-controlled Faradaic processes and surface-controlled capacitive processes, and to examine the interfacial charge transport dynamics of this material regarding charge storage/release processes. The relative contributions can be analyzed using following equation (Equation (1)):

I = aν^b^ (1)

where I is the current response (A); ν is the scan rate (V s^−1^); and a and b are the adjustable parameters in the above equation.

The value of b can be calculated to obtain the slope through plotting log I versus log ν using Equation (1). Generally, b = 0.5 indicates that the energy storage mechanism reveals a dominance of diffusion-controlled Faradaic behavior, whereas b = 1.0 signifies that surface-controlled capacitive behavior is dominant. In this work, the calculated value of b was near 0.5 for both redox peaks in the NiCo@3D Ni/SiC sample, indicating the dominance of diffusion-controlled pseudocapacitive kinetics [45]. In order to further explore the quantitative relationship between the surface- and diffusion-controlled contributions, Dunn’s equation (Equation (2)) was applied to further confirm the charge storage contributions in the NiCo@3D Ni/SiC sample [46,47]:

I(V) = k_1_ν + k_2_ν^1/2^ (2)

I(V)/ν^1/2^ = k_1_ν^1/2^ + k_2_ (3)

where I(V) is the current response (A) at an applied potential (V); ν is the scan rate (V s^−1^); and k_1_ and k_2_ are the adjustable parameters in the above equations.

The value of k_1_ and k_2_ can be calculated through plotting I(V)/ν^1/2^ versus ν^1/2^ using Equation (3). The obtained k_1_ and k_2_ values are the slope and intercept of the linear fitted line, respectively. Figure 5g presents the calculated charge storage percentage contribution in the NiCo@3D Ni/SiC sample at a scan rate of 5 mV s^−1^. The contribution percentages of diffusion-controlled Faradaic and surface-controlled capacitive behaviors are about 58% and 42%, respectively, which again demonstrate the synergetic effect from both the EDLC and pseudocapacitance (in which the diffusion-controlled Faradaic behavior dominates). In addition, regarding the surface and diffusion-controlled behaviors from the CV data at different scan rates, Figure 5h shows the percentage contribution of these two behaviors at various scan rates from 2 to 40 mV s^−1^ in the NiCo@3D Ni/SiC sample. The surface-controlled contribution gradually increases as the scan rates increases, originated from the limited diffusion of electrolyte ions at high scan rates [48].

Based on above results, the 3D Ni/SiC skeleton synthesized via a one-step binder-free hydrogen bubble template-assisted electrodeposition was interconnected with 3D Ni and SiC to form a 3D hierarchical structure, which provided numerous interior spaces in the 3D Ni/SiC network to promote the sequential growth of nickel–cobalt LDH nanosheets through electrodeposition, and the NiCo@3D Ni/SiC sample was obtained. The as-synthesized NiCo@3D Ni/SiC interconnected architecture exhibited a great supercapacitor performance compared with that of similar electrode materials based on the incorporation of semiconductor materials (Table 1) [49,50,51,52,53,54]. The interconnected architecture designs promoted suitable interior space to increase the exposure of the electroactive sites, which further ensured efficient ionic/electronic transport, significantly enhancing electrochemical supercapacitor performance. The improved electrochemical performance for NiCo@3D Ni/SiC is originated from the synergistic interaction between the pseudocapacitors (3D Ni skeleton and NiCo LDH) and EDLC (SiC nanopowders).

**Figure 6:**
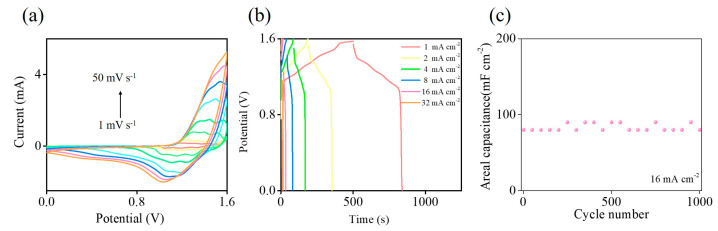
(**a**) CV (different scan rates from 1 to 50 mV s^−1^), (**b**) GCD (different current densities from 1 to 32 mA cm^−2^), and (**c**) cycling test (current density of 32 mA cm^−2^) of NiCo@3D Ni/SiC symmetric aqueous supercapacitor at different scan rates with a working voltage of 1.6 V in 1 M KOH solution.

To evaluate the practical use of the NiCo@3D Ni/SiC sample in supercapacitors, we assembled a symmetric aqueous supercapacitor device using two pieces of the as-synthesized NiCo@3D Ni/SiC sample in 1 M KOH electrolyte. Figure 6a presents measurements for the device within a 0 to +1.6 V potential window at scan rates from 1 to 50 mV s^−1^. Notably, the device exhibited a high operating voltage of 1.6 V without obvious polarization at a relatively slow scan rate, indicating high hydrogen and oxygen evolution overpotentials [55,56]. The CV curves maintained their shape despite increased scan rates, demonstrating the excellent capacitive behavior of the NiCo@3D Ni/SiC sample. To investigate the sample’s areal capacitance and rate capability, we conducted GCD measurements in the potential window of 0 to +1.6 V at current densities ranging from 1.0 to 32 mA cm^−2^ (Figure 6b). The GCD curves exhibited quasi-rectangular shapes with well-defined plateaus, indicating fast charge–discharge capability. Additionally, the symmetric aqueous supercapacitor device’s long-term capacitance stability was evaluated over 1000 cycles at a current density of 16 mA cm^−2^ using GCD measurements, as shown in Figure 6c. After 1000 cycles, the device retained nearly 100% of its initial specific areal capacitance. The GCD tests demonstrated the as-synthesized NiCo@3D Ni/SiC-based supercapacitor device’s excellent long-term cycling stability.

**Figure 7:**
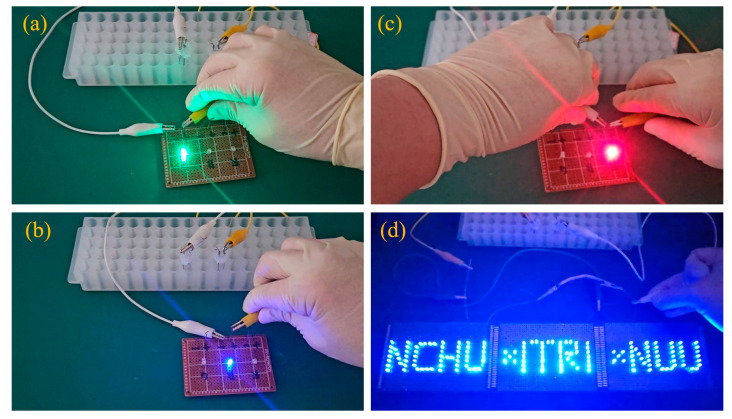
Photograph images of (**a**) green, (**b**) blue, (**c**) red, and (**d**) desired pattern LED lighting.

To further verify the practical usability of the as-synthesized NiCo@3D Ni/SiC-based supercapacitor, two symmetric aqueous supercapacitor devices were operated in series at 3.2 V to power light-emitting diodes (LEDs: green, blue, red, and a parallel National Chung Hsing University (NCHU) × Industrial Technology Research Institute (ITRI) × National United University (NUU) pattern formed with blue LEDs)). Figure 7 demonstrates that the two devices could easily power both single LEDs and the parallel blue LEDs. These results indicate that the as-synthesized NiCo@3D Ni/SiC-based symmetric supercapacitor device is viable for practical energy storage systems.

## 3. Experimental Section

### 3.1. Reagents

β-phase SiC nanopowder was obtained from Alfa Aesar (Ward Hill, MA, USA). Nickel(II) chloride hexahydrate (NiCl_2_·6H_2_O), nickel(II) nitrate hexahydrate (Ni(NO_3_)_2_·6H_2_O), cobalt(II) nitrate hexahydrate (Co(NO_3_)_2_·6H_2_O), and potassium hydroxide (KOH) were purchased from Sigma-Aldrich (St. Louis, MO, USA). All chemicals used were of analytical grade and were used as received without further purification. All solutions were prepared using a Milli-Q water purification system (Millipore, Milford, MA, USA). 

### 3.2. Preparation of Nickel–Cobalt Layered Double Hydroxide (LDH) Nanosheet-Decorated 3D Interconnected Porous Ni/SiC Skeleton

The 3D interconnected porous Ni/SiC skeleton was synthesized on a Ni wire (diameter of 0.25 mm) via one-step binder-free hydrogen bubble template-assisted electrodeposition (according to our previous research work) [57]. First, the Ni wire (4 cm) was sequentially cleaned ultrasonically in 1 M HCl, ethanol, and DI water for 15 min each to remove the contamination on the Ni substrate and then dried at 100 °C for the subsequent electrodeposition process. Then, electrodeposition was performed with a power supply system (high-voltage power supply) in a two-electrode system, with Ni wire as the cathode, Pt as the anode, and a 20 mL aqueous solution containing 0.5 mg mL^−1^ of SiC nanopowder, 2 M NH_4_Cl, and 0.2 M NiCl_2_·6H_2_O, which was used as the electrolyte for Ni/SiC electrodeposition to obtain the 3D interconnected porous Ni/SiC skeleton at an applied voltage of 6.0 V with an electrodeposition time of 600 s at room temperature. The resulting samples were designated as 3D Ni/SiC. Subsequently, the prepared 3D Ni/SiC skeleton was used as the substrate for nickel–cobalt layered double hydroxide (LDH) electrodeposition. As is typical, the electrodeposition was carried out in the electrolyte by dissolving 0.001 mole of Ni(NO_3_)_2_·6H_2_O and 0.001 mole of Co(NO_3_)_2_·6H_2_O in the 20.0 mL aqueous solution. Then, electrodeposition was performed in a three-electrode system, where the working electrode was the 3D Ni/SiC skeleton, an Ag/AgCl electrode was the reference electrode, and Pt wire was the counter electrode. A constant applied potential was implemented at −0.8 V vs. Ag/AgCl for 200 s at room temperature. After electrodeposition, the electrodes were taken out and washed with DI water, and then dried in an oven to remove the remaining reagents and collected for subsequent characterization. The obtained nickel–cobalt layered double hydroxide (LDH) nanosheet-decorated 3D interconnected porous Ni/SiC skeleton was designated as NiCo@3D Ni/SiC. For comparison, the 3D interconnected porous Ni skeleton (designated as 3D Ni) and nickel–cobalt LDH nanosheet-decorated 3D interconnected porous Ni skeleton (designated as NiCo@3D Ni) were also obtained by following the same procedure without the introduction of SiC, and the capacitive performance of the supercapacitor was compared with 3D Ni/SiC and NiCo@3D Ni/SiC. 

### 3.3. Characterization

The morphology was characterized using field-emission scanning electron microscopy (FESEM, JSM-7800F, JEOL, Akishima, Japan) and transmission electron microscopy (TEM, JEM-2010, JEOL, Tokyo, Japan). The chemical structure and composition was determined by X-ray photoelectron spectroscopy (XPS, PHI-5000 Versaprobe, ULVAC-PHI, Chigasaki, Japan). Raman spectra were characterized using an automated Raman spectrometer equipped with an argon laser excitation wavelength of 532 nm (Unidron, CL Technology Co., Ltd., New Taipei City, Taiwan). Electrochemical measurements were performed using either a two- or three-electrode system at room temperature by an electrochemical analyzer (Autolab, model PGSTAT30, Eco Chemie, Utrecht, The Netherlands). Cyclic voltammetry (CV) and galvanostatic charge discharge (GCD) were measured at scan rates of 1–50 mV s^−1^ and a current density of 1–32 mA cm^−2^ with a working potential window of 0.0 V to +0.5 V, respectively. The supercapacitor with the conventional three-electrode system comprised an electroactive material working electrode, a platinum wire counter electrode, and an Ag/AgCl (3 M KCl) reference electrode in an aqueous 1 M KOH electrolyte solution. In the two-electrode system, the symmetric supercapacitor device was assembled using two NiCo@3D Ni/SiC electrodes, which served as both the negative and positive electrodes inside the aqueous 1 M KOH electrolyte solution. 

## 4. Conclusions

In this study, we successfully prepared novel NiCo@3D Ni/SiC interconnected architectures composed of nickel–cobalt LDH nanosheets and SiC nanopowder within 3D interconnected porous Ni skeletons by two-step electrodeposition. The as-synthesized NiCo@3D Ni/SiC was used directly as an advanced electrode material, demonstrating high capacitive performance in supercapacitors. The interconnected design facilitates effective ionic and electrical transport between active materials and electrolytic ions by creating open interior spaces and ensuring effective electrical connections. This design increases the exposure of electroactive sites, promoting synergistic interactions between the pseudocapacitors (3D Ni skeleton and Ni–Co LDH) and EDLC (SiC nanopowders), thereby enhancing capacitive performance. The as-fabricated NiCo@3D Ni/SiC exhibited a high areal capacitance of 1565 mF cm^−2^ at a current density of 1 mA cm^−2^ and retained 90% of its capacitance after 1000 cycles. Symmetrical supercapacitor devices in series connection successfully powered commercial LEDs, indicating significant potential for the practical application of as-fabricated NiCo@3D Ni/SiC in supercapacitors.

## Figures and Tables

**Table 1:** Comparison of the supercapacitor performance in similar electrode materials.

Samples	Electrolyte	Current Density (mA cm^−2^)	Areal Capacitance (mF cm^−2^)	Reference
NiCo@3D Ni/SiC	KOH (1 M)	1	1565	This work
Reduced TiO_2_ NTs/Ni(OH)_2_	KOH (1 M)	0.75	306	[49]
MnO_2_/vertically oriented graphene/Si	Na_2_SO_4_ (1 M)	0.5	317	[50]
SiAN@PSS@GP@PSS-Pt	Na_2_SO_4_ (1 M)	1	719	[51]
SiNW/PEDOT@Pt/MnO_x_	Na_2_SO_4_ (1 M)	2	352	[52]
SiC@PANI	H_2_SO_4_ (1 M)	1	352	[53]
NiSi/SiC	KOH (1 M)	0.5	275	[54]

## Data Availability

Data is contained within the article.
